# Supplementary material for: Cross-biome microbial networks reveal functional redundancy and suggest genome reduction through functional complementarity
Source: Commun Biol. 2024 Aug 24;7:1046. doi: 10.1038/s42003-024-06616-5 (PMC11344793; doi:10.1038/s42003-024-06616-5)
Supplement: Supplementary file 2 — Supplementary Information [file 42003_2024_6616_MOESM2_ESM.pdf]

Supplementary Note 1: Calculation of aggregation scores assessing the propensity of pairs of taxa to appear together in the same samples

Null model

To assess the propensity of pairs of taxa to aggregate, we implemented the null model introduced by Navarro-Alberto & Manly (2009), already adopted by our group (Pascual-García *et al.* 2014) and summarized here for completeness.

The data consist of N taxa $i=1\ldots N$ observed at M locations $a=1\ldots M$, stored in the binary presence-absence matrix $X_{ia}\in\{0,1\}$. The null model expresses the probabilities $\pi_{ia}\equiv P(Y_{ia}=1)$ that generate random presence-absence matrices $Y_{ia}\in\{0,1\}$ as similar as possible to the observed one under the assumption that all Y_{ia} are independent (absence of interactions).

Navarro-Alberto and Manly proposed the parametrization $\pi_{ia}=1-\exp\left(p_i q_a\right)$, justified by assuming Poisson distributed species abundances. Unlike previous formulations, this formula guarantees that each π_{ia} takes values between 0 and 1. It expresses the $N\times M$ probabilities π_{ia} as a function of N taxon-specific parameters p_i plus$M$ location-specific parameters q_a, which we determine by maximizing the log-likelihood of the observed matrix given the model, $L=\sum_{ia}\left(X_{ia}\log(\pi_{ia})+(1-X_{ia})\log\left(1-\pi_{ia}\right)\right)$. We perform this maximization analytically, equating the first derivatives to zero by applying Newton's method with analytically computed gradients.

To take into account habitat preferences, we group locations a into environmental subtypes A according to the environmental classification from Pignatelli et al. (2009), and we adopt taxon-specific parameters $p_i(A)$ that depend on the subtype. This choice increases the number of parameters with respect to adopting environment-independent parameters p_i, but it reduces the chance that the inferred aggregation propensity is only based on shared habitat preferences.

Aggregation scores

In Pascual-García *et al.* (2014), we defined the bare aggregation score between two taxa *i* and *j* as minus the logarithm of the probability $P(n_{ij},M)$ that they co-occur at n_{ij} locations out of M under the null model. We performed an iterative computation, letting the number of locations vary from $m=0$ to M. The initial condition is $P(n_{ij}=0,0)=1$, $P(n_{ij}=1,0)=0$, and we update the probabilities as $P(n_{ij},m)=P(n_{ij},m-1)(1-\pi_{\mathrm{im}}\pi_{jm})+P(n_{ij}-1,m-1)(\pi_{\mathrm{im}}\pi_{jm})$.

However, the probability $P(n_{ij},M)$ is small for rare taxa whose taxon-specific parameter p_i is small, which tends to overestimate their aggregation scores. In order to reduce false positives, although at the possible expense to increase false negatives, here we compute the conditional probability to observe the companion taxon j given that the rare conditioning taxon i is present. We relabel i and j at each location such that $p_i(A)<p_j(A)$, i.e. the conditioning taxon i embodies the condition that is most difficult to fulfil, with the effect to increase the conditional probability. We compute the conditional probability of n_{ij} co-occurrences conditioned to the observed distribution of taxon i at all locations, denoted as $\{X_{ia}\}$, and we define the aggregation score between i and j as minus the probability that n_{ij} is equal or larger than the observed value, conditioned to the observed values of $\{X_{ia}\}$:

$$Aggr_{ij}=-\log\left[\sum_{n=n_{ij}^{obs}}^{M} P\left(n_{ij}=\mathrm{n}\,|\{X_{ia}\}\right)\right]=-\log\left[\sum_{n=n_{ij}^{obs}}^{M}\frac{P\left(n_{ij}=n,\{X_{ia}\}\right)}{P\left(\{X_{ia}\}\right)}\right]$$

The probability of taxon i at the denominator of the conditional probability is given by $P(\{X_{ia}\})=\prod_a [X_{ia}\pi_{ia}+(1-X_{ia})(1-\pi_{ia})]=\prod_a lik(X_{ia})$, i.e. it is the product of the likelihoods $lik(X_{ia})$ of the observed presence and absences of taxon i in each sample a. We compute the numerator of the conditional probability iteratively as

$$P(n_{ij},\{X_{ia}\},m)=$$
$$X_{\mathrm{im}}\pi_{\mathrm{im}}\left[P(n_{ij},\{X_{ia}\},m-1)(1-\pi_{jm})+P(n_{ij}-1,\{X_{ia}\},m-1)\pi_{jm}\right]+(1-X_{\mathrm{im}})(1-\pi_{\mathrm{im}})P(n_{ij},\{X_{ia}\},m-1).$$

For computational convenience, we compute one minus the probability that n_{ij} is smaller than the observed value, which can be computed faster. Likewise, we define the segregation score as minus the logarithm of the conditional probability that n_{ij} is equal to or smaller than the observed value.

Decoupling aggregation scores from cosmopolitanism

The mean aggregation score of a taxon is correlated with the number of samples in which the taxon is present, which we denote as its cosmopolitanism. To reduce this correlation, we transform the bare aggregation scores into *Z*-scores as follows. With the null model of the observed matrix we extract 100 random matrices, we compute their null model and, through it, we compute the bare scores S_{ij} for all pairs using the random matrix as it were the observed one. Finally, we obtain mean and standard deviation of these simulated scores S_{ij} and we use them to transform the observed score into a *Z*-score, $Z_{ij}=(S_{ij}^{obs}-\overline{S_{ij}})/\sigma(S_{ij})$, where S_{ij}^{obs} are the aggregation score obtained from the real data, and $\overline{S_{ij}},\sigma(S_{ij})$ are the mean and standard deviations of aggregation scores in the simulated data with a similar minimum cosmopolitanism as that of the real pair, obtained from a cubic spline fit. We transform the aggregation scores of the simulated data to *Z*-scores in a similar fashion, yielding a distribution of null *Z*-scores.

Thresholds

We use the distribution of null *Z*-scores obtained in the previous step to determine a *Z*-score threshold such that only 0.01% of the null Z-scores are higher than it (False Positive Rate = 0.0001). We then subtract this cut-off from the *Z*-scores obtained from the real observations in order to generate corrected *Z*-scores. Any pair of taxa with a positive corrected *Z*-score is deemed to significantly aggregate in our samples.

Supplementary Note 2: Comparison of genus-level core predictions using genomes from isolates and Metagenome-Assembled Genomes (MAGs) for different environments

This work relies heavily on a functional inference strategy in which the metabolic pathways associated to a given microbial genus are predicted from the functional annotations of all of the sequenced genomes from that genus. Similar functional inference strategies have been successfully applied before, with the most prominent example being the PiCRUST tool for the prediction of metagenome functions from marker genes (e.g. 16S rRNA gene) profiles (Douglas *et al.*, 2020), with 2942 citations at the time of writing this note (Google Scholar, accessed on 2024-04-05). However, there is a concern that this may be a source of biases, since a vast majority of the high quality reference genomes used as the basis for functional inference come from isolates, which may fail to capture the true environmental and functional diversity of the taxa whose functions are being inferred (Albright & Louca, 2023). An alternative to this would be to reconstruct Metagenome-Assembled Genomes (MAGs) from shotgun metagenomic sequences, in an attempt to capture the true functions associated to each taxa in the samples of interest without resorting to functional inference. MAGs are however often incomplete and can be biased due to sequencing depth and false positive effects (Browne *et* al., 2009; Meziti *et al.* 2021; Nunes de Rocha *et al.*, 2023).

To alleviate potential biases due to the use of genomes from isolates for functional inference, we restricted our analysis only to core genomes, that is, the metabolic pathways that were predicted in all of the available genomes from each genus. This restricts our analysis only to pathways that are conserved at the genus level, but is more robust as those are expected to be more conserved, regardless of whether the genomes used for inference are MAGs or isolates. To test these expectations, we sought to evaluate whether genus-level core predictions were similar when calculated using isolate genomes or MAGs. We chose to focus in the genus *Pseudomonas*, since

1) it has been extensively characterized, with thousands of genomes from hundreds of different species being available in public databases (Hesse *et al.*, 2018).

2) it is present in a wide range of environments (Spiers *et al.*, 2000; Silby *et al.*, 2011), and thus a good target to evaluate whether functional inference is affected by environmental biases.

3) it is metabolically versatile, with individual strains harboring mosaic genomes with customized genomic repertoires (Mathee *et al.*, 2008; Silby *et al.*, 2011). This complexity makes *Pseudomonas* a worst-case scenario for genus-level functional inference based on incomplete information, as inter-genome variability may result in different results depending on the number of genomes used for core genome estimation.

We downloaded 14464 high quality annotated *Pseudomonas* genomes from the proGenomes3 database (Fullam *et al.*, 2023). Those genomes had been annotated with KEGG Orthology terms using a common pipeline, and grouped into species-level clusters, which were then classified according to their occurrence into different habitats. Amongst these genomes, 14221 belonged to isolates, and 243 to high quality MAGs (CheckM: completeness > 90% and contamination < 5%; Parks *et* al., 2016; Fullam *et al.*, 2023). We then filtered out those genomes annotated with less than 1500 independent KO terms, which removed 79 genomes from isolates.

The resulting distribution of isolate genomes and MAGs across the different habitats considered in proGenomes3 is presented in **Supplementary Table SN2.1**). Genome size (measured as the number of unique KOs in the genome) was consistently smaller on MAGs than on isolate genomes (**Supplementary Table SN2.1**; **Supplementary Figure SN2.1**), with the size of MAGs being on average 83% of that of isolate genomes. This is noticeably smaller than the 90% MAG completeness threshold used in proGenomes3; it is unclear whether this obeys to a real biological cause (e.g. larger genomes being on average easier to isolate) or to errors in bioinformatics pipelines resulting in “high quality” MAGs that nonetheless are missing a larger fraction of genomic information than expected, a known issue further discussed in Meziti *et al*. (2021).

Habitat	Isolates		MAGs	
	**# Genomes**	**Average genome size (# KOs)**	**# Genomes**	**Average genome size (# KOs)**
**aquatic_habitat**	11780	2720	168	2245
**aquatic_sediment_mud _habitat**	9392	2777	124	2240
**soil_habitat**	13032	2701	189	2229
**host_associated**	13392	2695	134	2283
**aquatic_freshwater_ha bitat**	9968	2768	86	2276
**host_plant_associated**	10034	2767	23	2563
**disease_associated**	10031	2751	94	2244
**food_associated**	9226	2786	22	2590
**all**	14142	2677	243	2768

Supplementary Table SN2.1. Habitat distribution of the *Pseudomonas* genomes used to validate our functional inference approach.

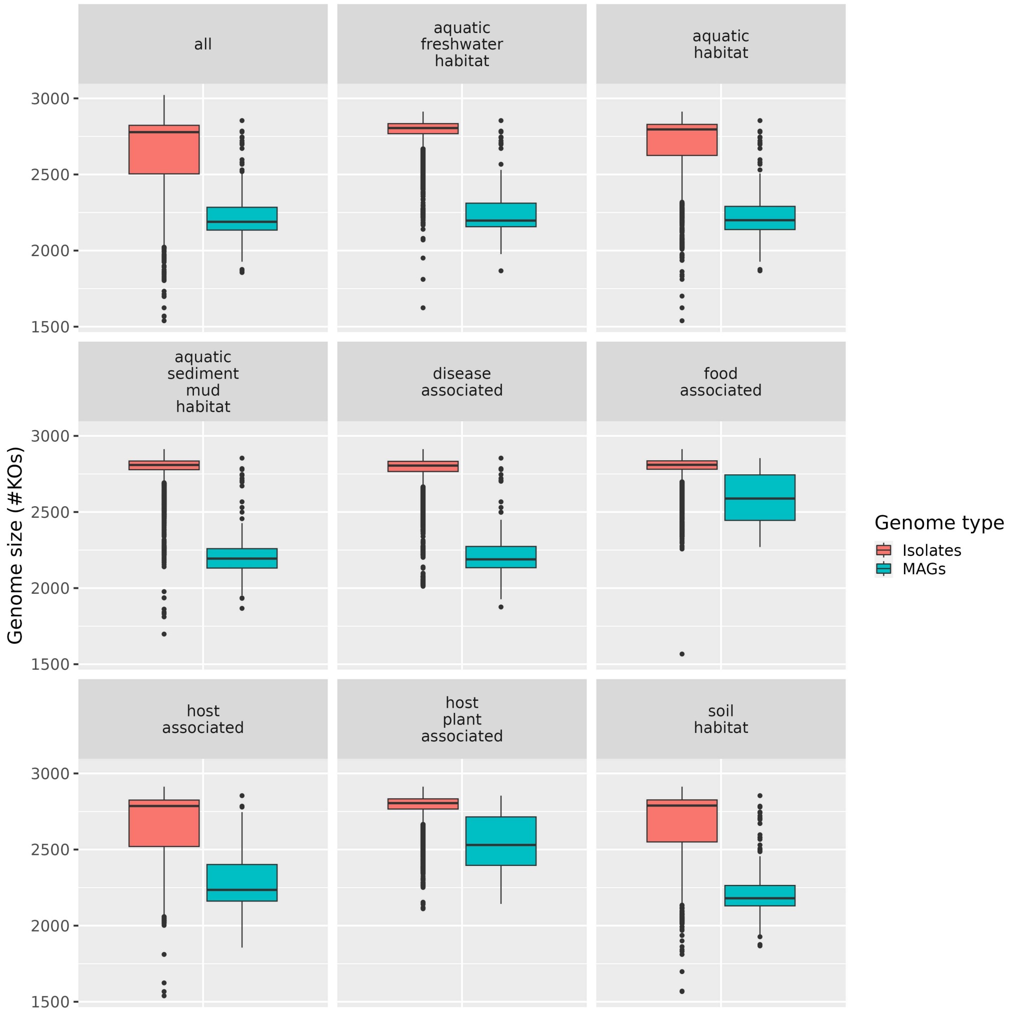

Supplementary Figure SN2.1. Genome size distribution (measured as number of unique KEGG Orthologs) of Pseudomonas isolate genomes and MAGs across different habitats.

We then used *mOTUpan* (Buck *et al.*, 2022) to estimate the core genome of *Pseudomonas* using the 14,142 available isolate genomes ("full core"). For each habitat, we also used *mOTUpan* to obtain core genome estimations after subsampling the number of isolate genomes and MAGs ("subsampled cores"; subsampling sizes ranging from 2 to 10000 genomes, 10 independent subsamples per subsampling size). m*OTUpan* was chosen for core genome estimation as it can provide robust estimations even when the input genomes are incomplete, as is typical of MAGs (Buck *et al.*, 2022). The cores obtained from MAGs were always smaller than the cores obtained from isolate genomes, regardless of the number of input genomes used for core calculation and of their source habitat (**Supplementary Figure SN2.2**), with the size of the cores obtained from MAGs being on average 81% of that of the cores obtained from isolate genomes. This result was consistent with our previous observation that *Pseudomonas* MAGs are generally smaller than isolate genomes (**Supplementary Figure SN2.1**).

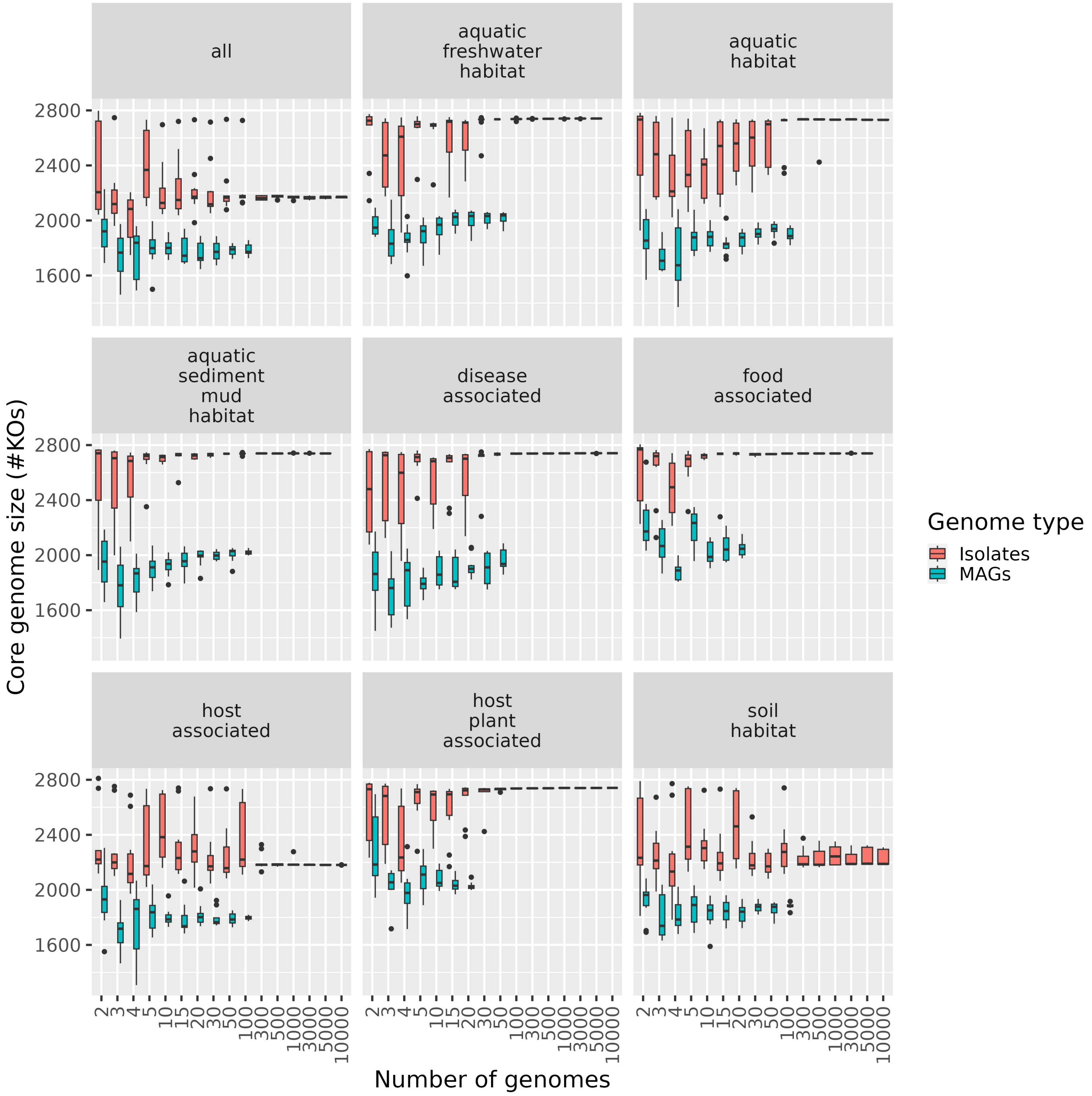

Supplementary Figure SN2.3. Core genome sizes (measured as the number of unique KEGG Orthologs) calculated using different subsamples of isolate genomes and MAGs (x axis). Each boxplot shows the distribution for 10 independent subsamplings.

In order to assess the robustness of genus-level core estimations, we compared the functional content of the “subsampled cores” (which aim to simulate a scenario in which the genus under study has a small number of available high quality genomes) to that of the “full core” (which we expect to be a reasonably close approximation to the real core genome of the genus). This procedure was performed independently for each habitat and subsampling size. With this, we aimed to answer the following questions:

1) Are genus-level core genome estimations different when using MAGs instead of isolate genomes?

2) What is the impact of the source habitat of the input genomes in genus-level core genome estimation?

3) What is the impact of the number of input genomes in genus-level core genome estimation?

The Jaccard similarity between the “full core” and the “subsampled cores” was consistently around 0.7 for the MAGs and 0.8 for the isolate genomes, regardless on the number of input genomes (**Supplementary Figure SN2.3**). The only exception were the isolate genomes coming from host associated and soil habitats, for which the Jaccard similarity to the “full core” approached 1 when the core genome was calculated using 300 genomes or more. This reflects the fact that the large majority of the *Pseudomonas* species included in the proGenomes3 database can be found in those habitats (**Supplementary Table SN2.1**) and thus had a larger influence in core-genome calculation. Regardless, the “subsampled cores” were good approximations of the genus-level core genome, even when calculated from a small number of MAGs. Furthermore, the lack of complete similarity between the core genomes as calculated with variable numbers of MAGs and the “full core” originating from 14,142 isolate genomes could be for the most part attributed to the MAGs cores being smaller on average and thus missing part of the content present in the “full core”. Indeed, on average 92% of the KEGG Orthologs present in the MAG cores were also present in the “full core” (**Supplementary Figure SN2.4**).

While *Pseudomonas* has a very large number of publicly available isolate genomes, this may not be the case for many of the genera included in this study, so the comparisons against the “full core” may not be fully representative. To account for this, we also performed direct comparisons between MAG and isolate cores calculated at the same subsampling size. The results were similar to those obtained when comparing “subsampled cores” from MAGs to the “full core” (**Supplementary Figure SN2.5**; **Supplementary Figure SN2.6**), with Jaccard similarity from MAG cores to isolate cores being on average 0.7, and the percentage of KEGG Orthologs from the MAG cores that were also contained in the isolate cores being on average 92%.

In summary, even for the highly variable *Pseudomonas* genus, the results obtained using isolate genomes and MAGs were comparable (>90% of the core obtained from the MAGs was also contained in the core obtained from the isolates), with most of the differences likely being related to the smaller average size of the MAGs. This difference in size may obey to biological causes or be an artifact of the bioinformatics pipelines used for MAG generation, and elucidating this is outside the scope of this work. Nonetheless, the similarity between the different approaches tested here strongly supports the notion that genus-level core genome calculations (such as as the ones used for functional inference in this work) are robust when using variable numbers of input genomes regardless of their type (MAGs vs isolate genomes) or source habitats.

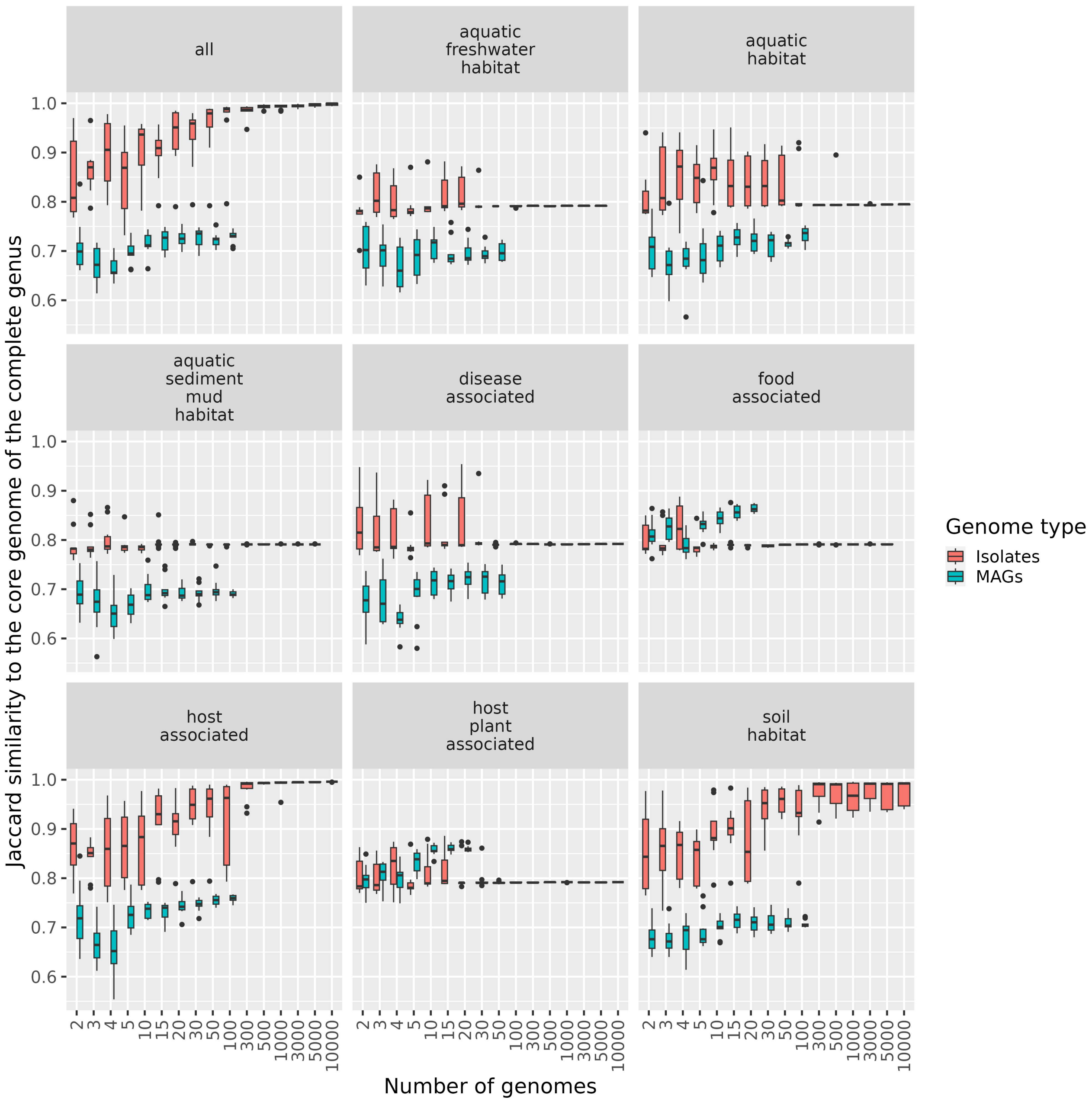

Supplementary Figure SN2.3. Jaccard similarities between the core genome of *Pseudomonas* when calculated using all the available isolate genomes and different subsamples of isolate genomes and MAGs (x axis). Each boxplot shows the distribution for 10 independent subsamplings.

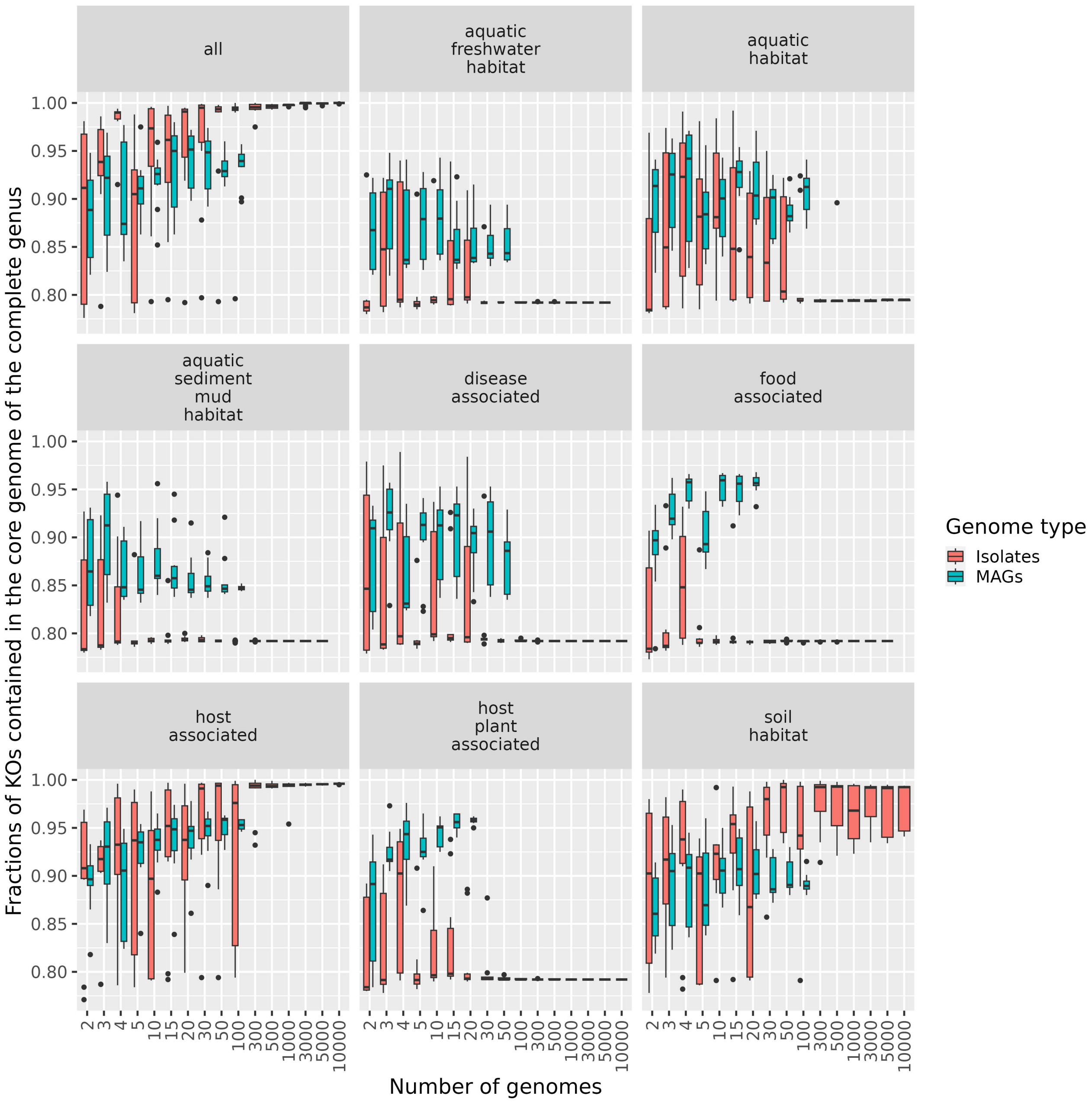

Supplementary Figure SN2.4. Fraction of KEGG Orthologs in core genomes calculated from different subsamples of isolate genomes and MAGs from Pseudomonas that were also present in the core genome when calculated with all the available isolate genomes. Each boxplot shows the distribution for 10 independent subsamplings.

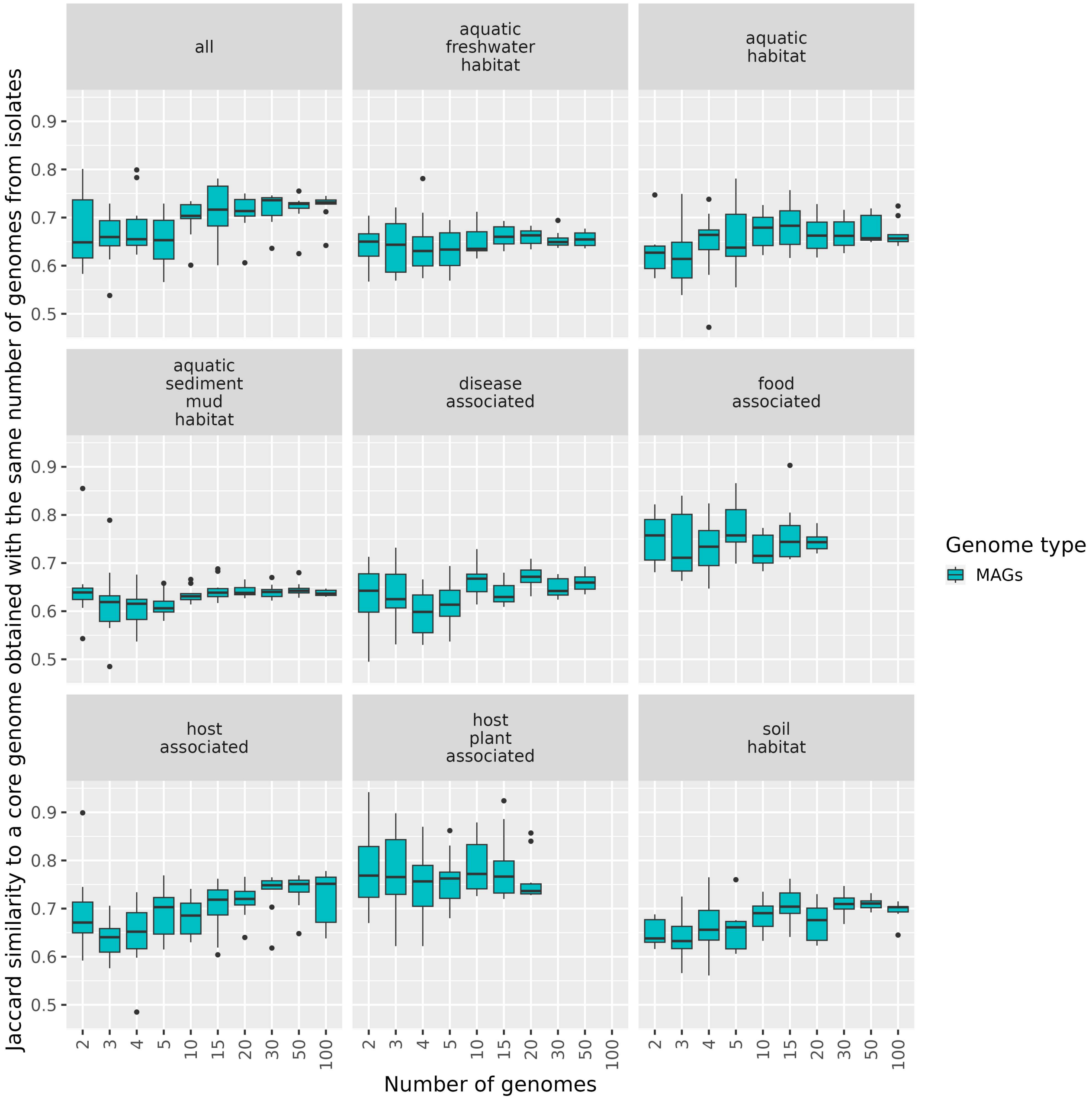

Supplementary Figure SN2.5. Jaccard similarities between the core genome of *Pseudomonas* when calculated different subsamples of MAGs (x axis) and the core genome when calculated using the same number of genomes from isolates. Each boxplot shows the distribution for 10 independent subsamplings.

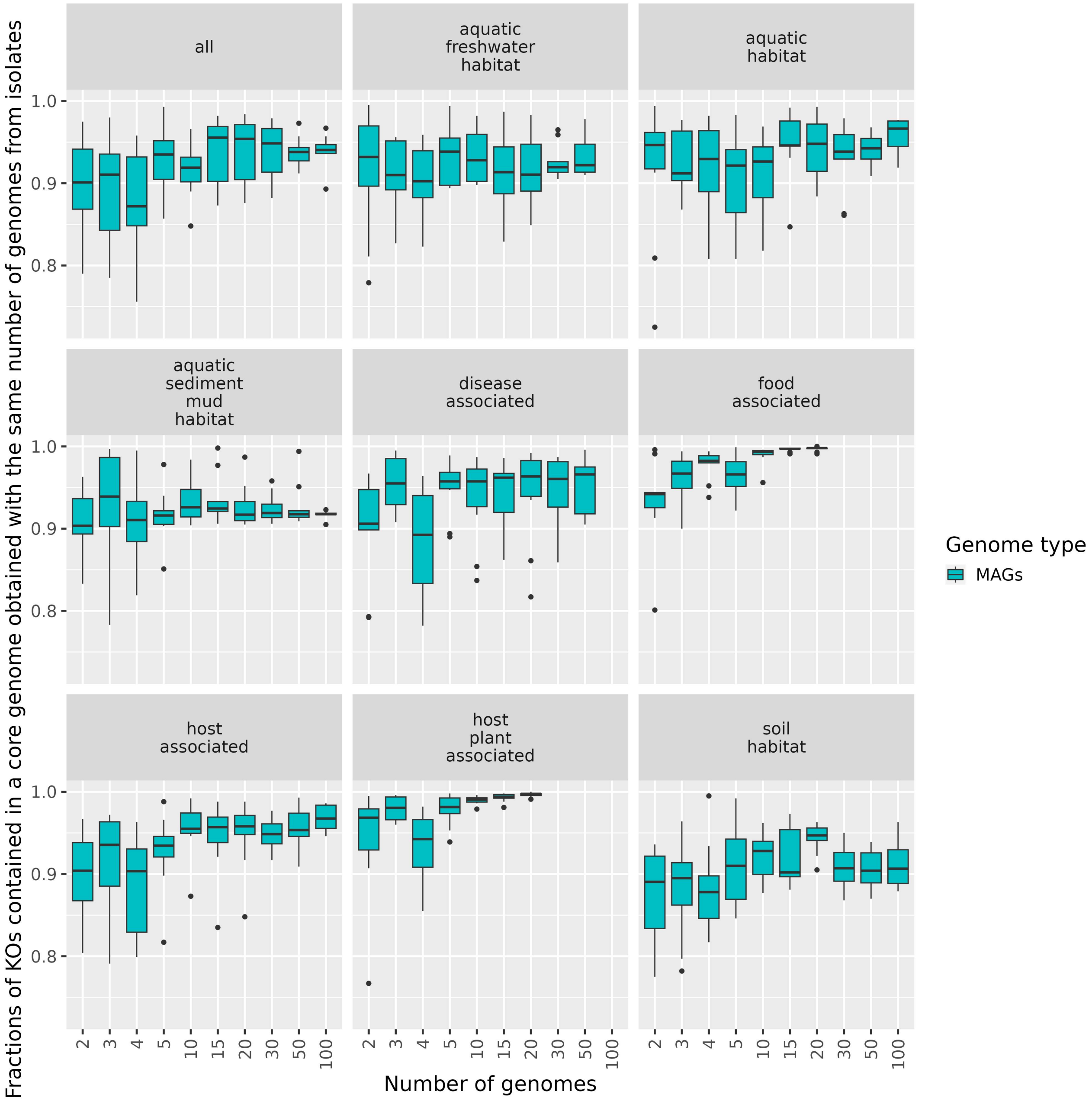

Supplementary Figure SN2.6. Fraction of KEGG Orthologs in core genomes calculated from different subsamples MAGs from Pseudomonas that were also present in the core genome when calculated using a similar number of isolate genomes. Each boxplot shows the distribution for 10 independent subsamplings.

Supplementary Figure 1. Average number of pathways per genome in environmental assemblages, using different size thresholds to discriminate between small and large assemblages. First panel (grey) shows the value for all the pathways, the other three (red, green, blue) show it for redundant, specific, and missing pathways. Number of assemblages per category and Wilcoxon p-value between small and large assemblages are indicated over the boxplots and under each panel respectively.

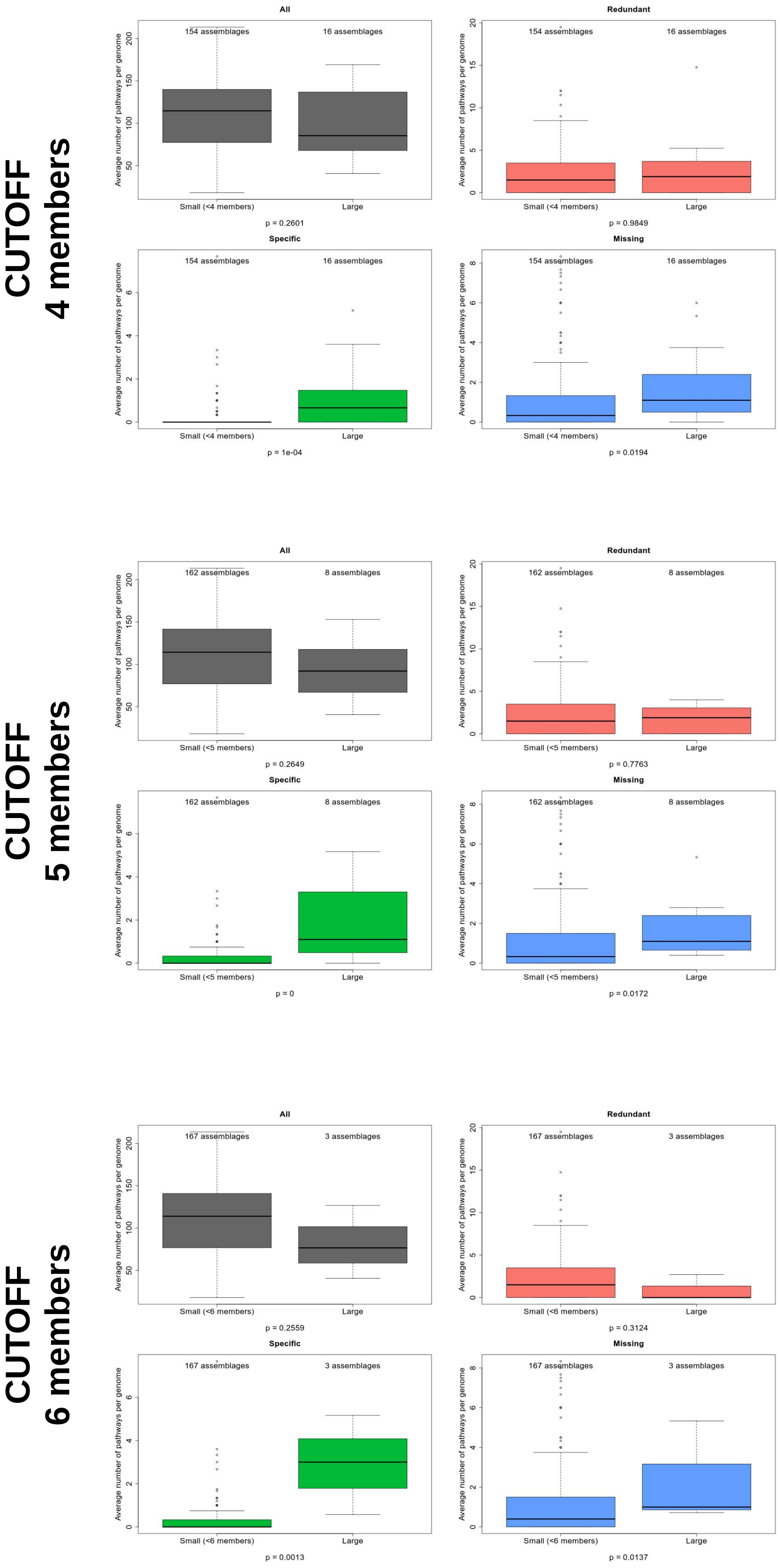

References

- Navarro-Alberto, J. A., & Manly, B. F. (2009). Null model analyses of presence–absence matrices need a definition of independence. Population ecology, 51(4), 505-512.
- Pascual-García, A., Tamames, J., & Bastolla, U. (2014). Bacteria dialog with Santa Rosalia: Are aggregations of cosmopolitan bacteria mainly explained by habitat filtering or by ecological interactions?. BMC microbiology, 14(1), 284.
- Pignatelli, M., Moya, A., & Tamames, J. (2009). EnvDB, a database for describing the environmental distribution of prokaryotic taxa. Environmental Microbiology Reports, 1(3), 191-197.Douglas, G. M., Maffei, V. J., Zaneveld, J. R., Yurgel, S. N., Brown, J. R., Taylor, C. M., ... & Langille, M. G. (2020). PICRUSt2 for prediction of metagenome functions. Nature biotechnology, 38(6), 685-688.
- Albright, S., & Louca, S. (2023). Trait biases in microbial reference genomes. *Scientific Data*, *10*(1), 84.
- Browne, P. D., Nielsen, T. K., Kot, W., Aggerholm, A., Gilbert, M. T. P., Puetz, L., ... & Hansen, L. H. (2020). GC bias affects genomic and metagenomic reconstructions, underrepresenting GC-poor organisms. *GigaScience*, *9*(2), giaa008.
- Meziti, A., Rodriguez-R, L. M., Hatt, J. K., Peña-Gonzalez, A., Levy, K., & Konstantinidis, K. T. (2021). The reliability of metagenome-assembled genomes (MAGs) in representing natural populations: insights from comparing MAGs against isolate genomes derived from the same fecal sample. *Applied and environmental microbiology*, *87*(6), e02593-20.
- Nunes da Rocha, U., Coelho Kasmanas, J., Toscan, R., Sanches, D. S., Magnusdottir, S., & Saraiva, J. (2023). Simulation of 69 microbial communities indicates sequencing depth and false positives are major drivers of bias in Prokaryotic metagenome-assembled genome recovery. *BioRxiv*, 2023-05.
- Hesse, C., Schulz, F., Bull, C. T., Shaffer, B. T., Yan, Q., Shapiro, N., ... & Loper, J. E. (2018). Genome‐based evolutionary history of Pseudomonas spp. *Environmental Microbiology*, *20*(6), 2142-2159.
- Spiers, A. J., Buckling, A., & Rainey, P. B. (2000). The causes of Pseudomonas diversity. *Microbiology*, *146*(10), 2345-2350.
- Silby, W., Winstanley, C., Godfrey, S., Levt, S. B., & Jackson R. W. (2011) *Pseudomonas genomes: diverse and adaptable*. *FEMS microbiology reviews* 35.4: 652-680.
- Mathee, K., Narasimhan, G., Valdes, C., Qiu, X., Matewish, J. M., Koehrsen, M., ... & Lory, S. (2008). Dynamics of Pseudomonas aeruginosa genome evolution. *Proceedings of the National Academy of Sciences*, *105*(8), 3100-3105.
- Fullam, A., Letunic, I., Schmidt, T. S., Ducarmon, Q. R., Karcher, N., Khedkar, S., ... & Mende, D. R. (2023). proGenomes3: approaching one million accurately and consistently annotated high-quality prokaryotic genomes. *Nucleic acids research*, *51*(D1), D760-D766.
- Parks, D. H., Imelfort, M., Skennerton, C. T., Hugenholtz, P., & Tyson, G. W. (2015). CheckM: assessing the quality of microbial genomes recovered from isolates, single cells, and metagenomes. *Genome research*, *25*(7), 1043-1055.
- Buck, M., Mehrshad, M., & Bertilsson, S. (2022). mOTUpan: a robust Bayesian approach to leverage metagenome-assembled genomes for core-genome estimation. *NAR genomics and bioinformatics*, *4*(3), lqac060.
