# Supplementary material for: Cross-biome microbial networks reveal functional redundancy and suggest genome reduction through functional complementarity
Source: Commun Biol. 2024 Aug 24;7:1046. doi: 10.1038/s42003-024-06616-5 (PMC11344793; doi:10.1038/s42003-024-06616-5)
Supplement: Supplementary file 3 — Description of Additional Supplementary Files [file 42003_2024_6616_MOESM3_ESM.pdf]

# Description of Additional Supplementary Files

File name: Supplementary Data 1

Description: Network in the Cytoscape format

File name: Supplementary Data 2
Description: General statistics on the network

File name: Supplementary Data 3

Description: Number of genomes from each genus in MetaCyc19

File name: Supplementary Data 4

Description: Fraction of genomes from each genus containing each MetaCyc pathway
